# Studies of the *in vitro* cytotoxic, antioxidant, lipase inhibitory and antimicrobial activities of selected Thai medicinal plants

**DOI:** 10.1186/1472-6882-12-217

**Published:** 2012-11-13

**Authors:** Chutima Kaewpiboon, Kriengsak Lirdprapamongkol, Chantragan Srisomsap, Pakorn Winayanuwattikun, Tikamporn Yongvanich, Preecha Puwaprisirisan, Jisnuson Svasti, Wanchai Assavalapsakul

**Affiliations:** 1 Program in Biotechnology, Faculty of Science, Chulalongkorn University, Phayathai Road, Bangkok, 10330, Thailand; 2 Laboratory of Biochemistry, Chulabhorn Research Institute, Vibhavadee Road, Bangkok, 10210, Thailand; 3 Department of Biochemistry, Faculty of Science, Chulalongkorn University, Phayathai Road, Bangkok, 10330, Thailand; 4 Department of Chemistry, Faculty of Science, Chulalongkorn University, Phayathai Road, Bangkok, 10330, Thailand; 5 Department of Microbiology, Faculty of Science, Chulalongkorn University, Phayathai Road, Bangkok, 10330, Thailand

**Keywords:** Antimicrobial, Antioxidant, Cytotoxic, Lipase inhibitory, Thai medicinal plants

## Abstract

**Background:**

Traditional folk medicinal plants have recently become popular and are widely used for primary health care. Since Thailand has a great diversity of indigenous (medicinal) plant species, this research investigated 52 traditionally used species of Thai medicinal plants for their *in vitro* cytotoxic, antioxidant, lipase inhibitory and antimicrobial activities.

**Methods:**

The 55 dried samples, derived from the medicinally used parts of the 52 plant species were sequentially extracted by hexane, dichloromethane, ethanol and water. These 220 extracts were then screened for *in vitro* (i) cytotoxicity against four cell lines, derived from human lung (A549), breast (MDA-MB-231), cervical (KB3-1) and colon (SW480) cancers, using the MTT cytotoxicity assay; (ii) antioxidant activity, analyzed by measuring the scavenging activity of DPPH radicals; (iii) lipase inhibitory activity, determined from the hydrolytic reaction of *p*-nitrophenyllaurate with pancreatic lipase; and (iv) antimicrobial activity against three Gram-positive and two Gram-negative bacteria species plus one strain of yeast using the disc-diffusion method and determination of the minimum inhibitory concentration by the broth micro-dilution assay.

**Results:**

The crude dichloromethane and/or ethanol extracts from four plant species showed an effective *in vitro* cytotoxic activity against the human cancer cell lines that was broadly similar to that of the specific chemotherapy drugs (etoposide, doxorubicin, vinblastine and oxaliplatin). In particular, this is the first report of the strong *in vitro* cytotoxic activity of *Bauhinia strychnifolia* vines. The tested tissue parts of only six plant species (*Allium sativum*, *Cocoloba uvifera*, *Dolichandrone spathacea*, *Lumnitzera littorea*, *Sonneratia alba* and *Sonneratia caseolaris*) showed promising potential antioxidant activity, whereas lipase inhibitory activity was only found in the ethanol extract from *Coscinum fenestratum* and this was weak at 17-fold lower than Orlistat, a known lipase inhibitor. The highest antimicrobial activity was observed in the extracts from *S. alba* and *S. caseolaris* against *Pseudomonas aeruginosa* and *Candida albicans*, respectively.

**Conclusion:**

The Thai medicinal plant *B. strychnifolia* is first reported to exert strong *in vitro* cytotoxic activities against human cancer cell lines and warrants further enrichment and characterization. The broad spectrum of the biological activities from the studied plant extracts can be applied as the guideline for the selection of Thai medicinal plant species for further pharmacological and phytochemical investigations.

## Background

Recent investigations into plants that have been the basis of traditional medicine for a long time have revealed a significant number of novel metabolites with potent pharmacological properties [1-3]. For example, the chemotherapeutic drug, pacitaxel (Taxol) was initially discovered from the bark of the Himalayan Yew tree by screening for anticancer activity in plant extracts [4]. Various types of plants have been used not only for dietary supplements but also as traditional folk treatments for many health problems [5]. Indeed, the long history of folk medicine demonstrates the potential of plants as sources of bioactive compounds [6]. Traditional medicine is widely used throughout Thailand [7], with both ready-made preparations and herbal drugs being used, and a number of these Thai medicinal plants have provided the foundation for modern pharmaceuticals and drug leads [8]. Thailand has a great diversity of indigenous (medicinal) plant species and hence is a potential source for bioactive compounds, including those with potential antitumor [7,9], antioxidant [10,11], anti-lipase (and so potential antiobesity) [9,12] and antimicrobial activities [13,14].

Since current pharmacological reagents are restricted by the increasing spread and evolution of resistance and/or their undesired side effects, and are often difficult to synthesize as the pure bioactive stereoisomer, investigations to explore novel drugs, or those that can act as templates for the development of new therapeutic agents appears imperative. Therefore, the aim of this study was to screen for potential *in vitro* cytotoxic, antioxidant, lipase inhibitory and antimicrobial activities from the crude extracts of the folk medicinally used parts of 52 species of Thai medicinal plants.

## Methods

### Chemicals and reagents

Ascorbic acid, chloramphenicol, dimethyl sulfoxide (DMSO), doxorubicin, etoposide, *p*-iodonitrotetrazolium violet*, p*-nitrophenyllaurate (*p*-NPL) vinblastine, oxaliplatin, porcine pancreatic lipase (PPL; Type II: from porcine pancreas), 2,2-diphenyl-1-picryhydrazyl (DPPH) and 3-(4,5-dimethylthiazol-2-yl)-2,5-diphenyltetrazolium bromine (MTT) were purchased from Sigma (USA). Amphotericin B was purchased from Bristol-Myers Squibb (France). Orlistat (Xenical®) was purchased from Roche (Italy). Cell culture media and antibiotics were purchased from Gibco (USA). Fetal bovine serum (FBS) was purchased from Hyclone (USA). All other chemicals were of analytical grade.

### Plant materials

Fifty two types of Thai medicinal plants were selected based on traditional medicinal uses. These plants were grown in Pom Phra Chulachomklao Mangrove Forests, Phra Chulachomklao Fort, Samut Prakarn (Additional file 1: Table S1). The taxonomic identification was done by the members of the Mangrove Forest Restoration and Regeneration Project during 2004 to 2008 from the Metropolitan Electricity Authority, using the available taxonomic key with the aid of the relevant literature (e.g. the ecology of mangrove plants [15] and flora of Thailand). The specimens, collected during August to October 2010, were dried, ground into a fine powder and then extracted as previously described in Mothana *et al.*[16] with minor modification. Twenty grams of the sample powder was sequentially extracted with hexane, dichloromethane (DCM) and ethanol (300 mL each), respectively, by Soxhlet extraction for 8 h. The residues were dried overnight and then extracted with 300 mL water in a shaking water-bath at 60°C for 8 h. The obtained crude hexane, DCM and ethanol extracts were evaporated to dryness on a rotary evaporator while the crude water extract was freeze dried. The dried samples were then stored at −20°C until use.

### *In vitro* cytotoxic activity assay

The *in vitro* cytotoxic activity of the crude extracts was determined from the mitochondrial activity of cell lines which represent the number of viable cells after the treatment, by using the MTT cytotoxic assay as previously described [17] on four different human cell lines in tissue culture. The non-small cell lung adenocarcinoma (A549) and breast cancer (MDA-MB-231) cell lines were purchased from the American Type Culture Collection (Manassas, VA, USA). The cervical (KB3-1) and colon (SW480) cancer cell lines were kindly provided by Professor Gottesman (Laboratory of Cell Biology, National Cancer Institute, National Institute of Health, MD, USA) and Dr. Chanida Vinayanuwattikun (Faculty of Medicine, Chulalongkorn University), respectively.

Cell suspensions in complete medium (CM) (either RPMI (A549 and SW480) or DMEM (MDA-MB-231 and KB3-1), supplemented in both cases with 10% (v/v) FBS, 100 units/mL penicillin and 100 μg/mL streptomycin), were seeded into each well of a 96-well plate (5 x 10^3^ cells per well) and incubated at 37°C with 5% (v/v) CO_2_. After 24 h, the crude extracts at five different concentrations in DMSO (two-fold serial dilutions from 100 to 6.25 μg/mL) dissolved in the respective CM were then added into the wells and further incubated for 72 h. Thereafter, the media in the wells were removed and replaced with fresh CM containing 5 mg/mL MTT and incubated at 37°C for 2 h to allow the formation of the insoluble formazan crystal by the mitochondrial active (viable) cells. The media were then removed, 100 μL DMSO was added to lyse the cell membranes and solubilize the formazan crystals and the absorbance was measured at 550 nm using a Biochrom Asys UVM 340 Microplate Reader (Holliston, MA, USA). The percentage of cell survival was calculated from Eq. (Eq. 1).

(Eq.1) $$\%\;\text{Cell survival}\;=\;\left({\text{OD}}_{\text{T}}/{\text{OD}}_{\text{C}}\right)\;\times \;100$$

where OD_T_ and OD_C_ are the mean absorbance of the treated and the control cells, respectively.

The concentration of the extract which caused a half maximal inhibition of cell proliferation (IC_50_), as determined by the MTT assay, was obtained from a semilog plot of the crude extract concentrations against the percentage of cell survival. Etoposide (200–0.39 μg/mL), doxorubicin (50–0.1 μg/mL), vinblastine (100–0.2 μg/mL) and oxaliplatin (100–0.2 μg/mL) were used as the specific positive controls for the A549, MDA-MB-231, KB3-1 and SW480 cell lines, respectively.

### DPPH radical scavenging (antioxidant activity) assay

The DPPH free radical scavenging assay was used for the evaluation of the antioxidant activity of the crude extracts, as previously described [16]. The dried crude hexane, DCM, ethanol and water extracts were each dissolved to five different concentrations in ethanol (10, 50, 100, 500 and 1000 μg/mL). The reaction mixture, containing 100 μL of the desired extract concentration in ethanol, 25 μL of 1 mM DPPH and 75 μL of ethanol were added into a 96-well plate and incubated at 37°C for 30 min. The absorbance at 517 nm was then monitored from the yellowish solution in a Biochrom Asys UVM 340 Microplate Reader. The DPPH radical scavenging activity was then calculated from Eq. (Eq. 2).

(Eq.2) $$\begin{array}{c} \%\;\text{of DPPH radical scavenging activity}\\ \;=\left[\left({\text{A}}_{0}-\left({\text{A}}_{1}-{\text{A}}_{2}\right)/{\text{A}}_{0}\right)\right]\;\times \;100 \end{array}$$

where A_0_, A_1_ and A_2_ are the absorbance of the DPPH (no crude extract), the crude extract with DPPH and the crude extract without DPPH, respectively.

The concentration which caused a half-maximal reduced DPPH radical level (EC_50_) was then determined. Extracts with an EC_50_ < 10 μg/mL were considered as having a high level of antioxidant activity [18]. Ascorbic acid (10, 50, 100, 500 and 1000 μg/mL) was used as a positive control.

### Determination of the lipase inhibitory activity

The lipase inhibitory activity of the crude extracts was estimated from the ability to inhibit the *in vitro* porcine pancreatic lipase activity as previously described [19] with slight modification. Briefly, the dry crude ethanol and water extracts were dissolved in 50 mM Tris–HCl pH 8.5 containing 50% (v/v) DMSO to a concentration of 50 mg/mL. The assay mixture contained 10 μL of one of five different concentrations (two-fold serial dilutions from 2.5 to 0.156 mg/mL) of the crude extracts, 12 μL of 20 mg/mL of PPL (type II) in 50 mM Tris–HCl pH 8.5 and 10 μL of 5.1 mM *p*-NPL in ethanol. The lipase activity was determined from the hydrolysis of *p*-NPL by measuring the absorbance at 410 nm of the *p*-nitrophenol product formed at 37°C for 5 min using a FLUOstar OPTIMA micro reader (BMG LABTECH, Offenburg, Germany). The lipase inhibitory activity was expressed as the percentage of the decrease in A_410_ when PPL was incubated with the crude extracts compared to the negative control (solvent only), and was calculated from Eq. (Eq. 3).

(Eq.3) $$\%\;\text{of enzyme inhibition}=\left[\text{E}-\text{T}/\text{E}\right]\times 100$$

where E and T are the absorbance of the reaction without and with the crude extract, respectively.

The concentration of the extract which caused a half maximal inhibition of the lipase activity (IC_50_) was obtained from a semilog plot of the crude extract concentrations against the percentage of enzyme inhibition. Orlistat (250–0.49 μg/mL) was used as the positive control.

### Antimicrobial assay

Three Gram-positive (*Bacillus subtilis* (MCCU0351), *Micrococcus luteus* (MCCU0350) and *Staphylococcus aureus* (MCCU0352)) and two Gram-negative (*Escherichia coli* (MCCU0349) and *Pseudomonas aeruginosa* (MCCU0359)) bacterial strains, plus the yeast (*Candida albicans*) were used for the antimicrobial screening.

The antimicrobial activities of the crude ethanol and water extracts were determined using the disc-diffusion and broth micro-dilution assays [16]. For the disc-diffusion assay, the various microbes were spread at 1.5 x 10^7^ cells/plate on 9-cm diameter nutrient agar petri dishes. Sterile filter paper discs of 6 mm in diameter (Whatman, cat. NO. 1442125, lot 577125) were impregnated with 2 mg of the crude extract dissolved in 5% (v/v) ethanol, dried and then placed on the surface of the spread agar plates. They were kept for 2 h in a 4°C refrigerator to enable pre-diffusion of the crude extracts and then further incubated at 37°C for 18 h, except for *M. luteus* (room temperature for 48 h) and *C. albicans* (30°C for 48 h) [20]. At the end of the incubation, the antimicrobial activity was evaluated by measuring the diameter of the inhibition zone. An inhibition zone of 10 mm in diameter or more was regarded as representing a high antimicrobial activity. Chloramphenicol (20 μg/disc) and amphotericin B (40 μg/disc) were used as positive controls for the bacteria and yeast, respectively.

The crude extracts that revealed a positive antimicrobial activity in the disc-diffusion assay were then evaluated by the broth micro-dilution assay to determine the minimal inhibitory concentration (MIC) against the microorganisms as previously reported [16], except with minor modifications. Within sterile 96-well plates, two-fold serial dilutions of the selected crude extracts were prepared (in duplicate) in the appropriate broth containing 5% (v/v) DMSO to produce a concentration range of 1,000 to 7.8 μg/mL. The bacterial cell suspension, at 0.5 McFarland standards (approximately 1.5 × 10^8^ colony forming units/mL) [21] was added into each well, except for the blank wells which served as extract and media sterility controls. Control cultures for bacterial growth without the crude extract were also included in each plate. The plates were then incubated at 37°C for 18 h, where after 20 μL of a 0.04% (w/v) *p*-iodonitrotetrazolium violet solution was then added to each well and incubated for 30 min. A change in color from yellow to pink, indicating the reduction of the dye from the bacterial growth, was observed. The MIC of the crude extract was determined from the lowest concentration at which no growth of microorganism, as determined by the color change, was observed. Similarly, two-fold dilutions of chloramphenicol (250–7.81 μg/mL) or amphotericin B (500–15.63 μg/mL) were used as a positive control for the bacteria and yeast, respectively.

## Results

Additional file 1: Table S1 lists the ethnobotanical data of the investigated plant species illustrating their botanical names, the part(s) of the plant used for extraction, the traditional folk medicinal uses of these plant tissues, and the yield obtained from each extract (as % by dry weight of the total dry weight). A total of 220 extracts, representing 52 plant species (since two tissue parts were used for each of *Bauhinia strychnifolia*, *Moringa oleifera* and *Solanum trilobatum*), were screened for *in vitro* cytotoxic, antioxidant, lipase inhibitory and antimicrobial activities.

### *In vitro* cytotoxic activity

Of the 52 plant species assayed, the extracts from only four species (*B. strychnifolia* vines, *Coscinium fenestratum* stems*, Eurycoma longifolia* roots and *Kalanchoe pinnata* leaves) displayed potential *in vitro* cytotoxicity against the four human cancer cell lines tested (Additional file 1: Table S2).

In terms of the deduced IC_50_ values, the crude water extracts had only a weak (> 20 μg/mL) to essentially no activity in all cases, but for the other less polar extracts they varied between the cell lines, plant species and solvents used in the extraction. Nevertheless, typically the crude DCM and ethanol extracts were more active suggesting the bioactive components have a moderate to low polarity.

Overall, the vine extracts from *B. strychnifolia* were the most effective against three of the four human cancer cell lines. However, it was still effective against the fourth (SW480) cell line. The highest cytotoxic activity (IC_50_ of 0.28 μg/mL) was obtained against the MDA-MB-231 cell line by the crude hexane extract of *B. strychnifolia* vines, with the crude DCM extract being strongly cytotoxic against the A549 and KB3-1 cell lines (IC_50_ value of 1.16 and 1.86 μg/mL, respectively). In fact, this is the first report of the *in vitro* cytotoxic activity of this interesting plant against human transformed cell lines*.*

For the remaining three plant species with positive *in vitro* cytotoxicity, the crude DCM and ethanol extracts of *K. pinnata* leaves showed a better overall *in vitro* cytotoxicity than the other two plant species, although these *K. pinnata* leaf extracts were not that effective against the A549 cell line. The crude DCM and ethanol extracts from *C. fenestratum* stems were effective against the KB3-1 cell line (IC_50_ values of 3.25 and 2.18 μg/mL, respectively). Although the extracts from *E. longifolia* roots were in general the least effective of the four positive plant species, its DCM and ethanol extracts were strongly cytotoxic against the MDA-MB-231 cell line (IC_50_ values of 1.6 and 1.2 μg/mL, respectively).

### Antioxidant and lipase inhibitory activities

With respect to the antioxidant activity, an EC_50_ value of less than 10 μg/mL in the DPPH radical scavenging assay is generally considered to be effective in this work. However, the positive control of ascorbic acid under these assay conditions was outside this limit (EC_50_ = 12 ± 1.29 μg/mL). Of the 220 plant extracts, only some of the crude water and ethanol extracts, but not the hexane and DCM extracts, from six plant species (see below) revealed potential DPPH radical scavenging activity. Therefore, it can be speculated that the principal bioactive components are relatively polar compounds and different to those with cytotoxic activities.

Of the 110 crude ethanol and water extracts evaluated from the 52 plant species, extracts from only six plant species were found to have an effective DPPH radical scavenging activity, ranging in the order (lowest to highest EC_50_ value) of *Sonneratia caseolaris* leaves, *Coccoloba uvifera* leaves, *Sonneratia alba* leaves, *Lumnitzera littorea* leaves, *Allium sativum* bulbs and *Dolichandrone spathacea* leaves (EC_50_ values of 1.92 ± 0.38, 3.08 ± 1.01, 3.27 ± 0.53, 4.00 ± 0.25, 4.23 ± 0.67 and 5.17 ± 0.29 μg/mL, respectively).

With respect to the crude water extracts, three (*S. alba*, *S. caseolaris* and *L. littorea*) of the six plant species with effective ethanol extracts also had effective DPPH radical scavenging activity (EC_50_ values of 6.43 ± 2.29, 7.25 ± 1.52 and 7.27 ± 0.64 μg/mL, respectively), but no other extracts were effective. Although the water extracts were less effective than the corresponding ethanolic ones, it should be born in mind that these are derived from sequential (ethanol before water), potentially non-exhaustive extractions that will vary in their compositions and so it cannot be inferred that they represent different component(s) or specific activities.

With respect to the lipase inhibition, the crude ethanol extract from *C. fenestratum* was the only one found to display effective activity. Nevertheless, this was weak (IC_50_ value of 160 ± 0.02 μg/mL), compared to that for the positive control of Orlistat (IC_50_ value of 9.25 ± 1.25 μg/mL).

### Antimicrobial activity

Using the disc-diffusion assay and taking an inhibition zone of ≥10 mm diameter as the indication of a strong antimicrobial activity [22], from the 110 crude ethanol and water extracts screened from 52 plant species, those from only four plant species were found to exhibit a strong antimicrobial activity (Additional file 1: Table S3). The ethanol extracts of *C. fenestratum* stems showed the broadest range of activity, inhibiting all of the three tested Gram-positive bacteria (*S. aureus, B. subtilis* and *M. luteus*), and one (*E. coli*) of the two Gram-negative bacteria as well as the yeast strain (*C. albicans*). However, the crude ethanol extract from *Anacardium occidentale* leaves and *S. caseolaris* leaves were also effective against *E. coli*.

For the aqueous extracts, that from *S. alba* leaves was found to inhibit one (*M. luteus*) of the three tested Gram-positive bacteria, both tested Gram-negative bacteria (*E. coli* and *P. aeruginosa*) and the yeast strain (*C. albicans*), whilst that from *S. caseolaris* leaves could inhibit *E. coli* and *C. albicans*. The extracts from *A. occidentale* leaves, therefore, appeared to potentially be specific for Gram-negative bacteria.

To obtain the MIC of the crude extracts, the broth micro-dilution assay was utilized to screen those crude water and ethanol extracts that were positive by the disc-diffusion assay. The results (Additional file 1: Table S3) showed that the crude ethanol extract of *C. fenestratum* stems inhibited the three tested Gram-positive and one (*E. coli*) of the two tested Gram-negative bacteria strains plus *C. albicans* equally and effectively (MIC values of 500 μg/mL), whilst the only other extract to be active against Gram-positive bacteria (water extract of *S. alba* on *M. luteus*) also had a MIC value of 500 μg/mL. However, a lower MIC was obtained against the Gram-negative bacteria *P. aeruginosa* and the yeast *C. albicans* from the aqueous extracts of *S. alba* and *S. caseolaris* (125 μg/mL). These MIC values are typically higher than those for the positive control of chloramphenicol for bacteria (MIC of ~8 to 32 μg/mL, except for *P. aeruginosa* at 125 μg/mL) but broadly comparable to that for amphotericin B for the yeast (MIC of 250 μg/mL). However, these crude extracts are likely to contain many non-bioactive compounds (in mass) and so the actual specific activities after enrichment would potentially be higher.

## Discussion

### *In vitro* cytotoxic activity

According to the United States National Cancer Institute plant screening program, a plant extract is generally considered to have an active cytotoxicity effect if the IC_50_ value following incubation between 48 to 72 h, is 20 μg/mL or less [23].

The relevant tissue(s) (in terms of the tissue(s) used in folk medicine) from 52 species of medicinal plants in Thailand were screened as their crude hexane, DCM, ethanol and water extracts for *in vitro* cytotoxic activity against four human transformed (cancer) cell lines. The highest cytotoxic activity was obtained from the crude hexane extract obtained from the vines (but not leaves) of *B. strychnifolia* against the MDA-MB-231 cell line. Moreover, this extract also had potential strong activity against the KB3-1 cell line, whilst the crude DCM extract exhibited cytotoxic activity against the A549 and KB3-1 cell lines. From the results, the derived IC_50_ values for the crude hexane and DCM extracts from the vines of *B. strychnifolia* are not that different on each cell line from the respective positive controls (etoposide, doxorubicin, vinblastine and oxaliplatin). Assuming no strong synergy among all components in the extract, it can be implied that the bioactive component(s) in these fractions could be far more potent than the standard reference drugs and so merits their enrichment and further characterization.

Clinically, *B. strychnifolia* has been applied for the treatment of human food poisoning diarrhea [24] and also in 2011 as an anti-HIV-1 agent [25]. Nevertheless, the antitumor activity has never been reported. As a consequence, this work appears to be the first report on the antitumor activity of *B. strychnifolia*.

The crude DCM extract of *C. fenestratum* showed *in vitro* cytotoxicity against the KB3-1 cell line, which is consistent with the reported antiproliferative activity against the human colorectal carcinoma (HCT-116) cell line [26], where it apparently induces expression of the peroxisome proliferator-activated receptor γ and pro-apoptotic genes.

The crude DCM and ethanol extracts of *E. longifolia* roots exhibited cytotoxic activity against the MDA-MB-231 cancer cell line, which has been reported previously on the human breast cancer cell line MCF-7 where the inhibition was linked to the induction of apoptotic cell death [27,28]. In addition, from nearly 65 compounds isolated from the roots of *E. longifolia*, eight were found to demonstrate strong cytotoxicity towards the human lung cancer (A549) cell line and some of these were also strongly cytotoxic against the MCF-7 cell line [29]. Thus, it appears that the crude alcohol extracts from *E. longifolia* roots may exhibit a preferential or specific cytotoxicity against breast cancer.

The bufadienolide isolated from the methanol extract of *K. pinnata* has been reported to be a potential cancer chemotherapeutic agent since it inhibits the tumor promoting activity of Epstein-Barr Virus [30]. Here, we found that the crude ethanol extract from the leaves of this plant inhibited the growth of the KB3-1 cell line, which was developed from human papilloma virus infected cells. Hence, it is possible that the ethanol extracts affect the regulation of some viral proteins that control cell division.

Overall, the crude hexane and DCM extracts were more active than the corresponding aqueous and ethanol ones, suggesting that the active compound(s) against the cell lines in these plants are of low polarity. In addition, as mentioned before, since these are crude extracts and may contain many non-active components then the IC_50_ values reported here may in fact be far higher than those of the actual bioactive component(s) in the extracts, assuming no strong synergy between different components. Therefore, these results support that the bioactivity-guided enrichment of these fractions is merited.

### Antioxidant and lipase inhibitory activity

With respect to the antioxidant activity, the crude ethanol and water extracts from only six of the 52 investigated plants showed any effective free radical scavenging activity in the DPPH assay when compared to that of the ascorbic acid reference standard. The hexane and DCM extracts did not exert any detectable antioxidant activity in this study. This result is similar to the study on Fenugreek seeds (*Trigonella foenum-graecum*) which showed that the highest antioxidant activity was found in the ethanol and methanol extracts followed by the aqueous extract with only low activities in the hexane and DCM extracts [31]. Moreover, most of the 52 plants investigated in this study naturally occur (and were obtained from) within mangrove forest areas, which are typically rich sources of phenolic compounds, such as flavonoids [32], to protect the plants from UV radiation [33,34]. As a matter of fact, a linear relationship between the flavonoid content and the antioxidant activity has already been reported [35].

With respect to the lipase inhibitory activity, only the crude ethanol and water extracts were evaluated. The low polarity compounds including the natural lipids in the tissue(s) could be extracted by hexane and DCM at sufficiently high concentrations as to interfere in the assay by acting as alternative but unquantified substrates for the lipase providing false positives and potentially masking genuine weak and moderate positives. However, all but one of the 110 water and ethanol extracts were found to be inactive. The exception was the ethanol extract from *C. fenestratum* stems that exhibited a weak lipase inhibitory activity (IC_50_ value of 160 μg/mL) that had a 17.3-fold lower IC_50_ value than that for Orlistat, a known lipase inhibitor. Nevertheless, it is a crude extract, and so the actual IC_50_ of the active component(s) may be significantly higher. The lipase inhibitory activity from this plant (the first report) gives a suggestion of the potential to screen for novel plant compounds with anti-lipase activity. These may be of clinical dietary use in countering the problems of human obesity. Thus, it may be interesting for further studies to fractionate the crude hexane and DCM extracts to remove the natural lipid content and screen the other fractions for anti-lipase activity.

### Antimicrobial activity

Unfortunately, the crude hexane and DCM extracts, although soluble in DMSO were not soluble in the 5% (v/v) DMSO-nutrient broth media used for the broth micro-dilution assay, where higher DMSO levels are themselves inhibitory in the assay. Therefore, only the 110 crude ethanol and water extracts were screened for antimicrobial activity. Significant antimicrobial activities (inhibition zones ≥ 10 mm) were obtained from the extracts of only four plant species, with MIC values of ≤ 500 μg/mL (as determined by subsequent broth assays).

Of the four plants with antimicrobial activity, the crude ethanol extract of *C. fenestratum* stems displayed a broad range of antimicrobial effects against all tested microorganisms except *P. aeruginosa*. Moreover, it has been previously reported to inhibit the growth of *Propionibacterium acnes* and *Staphylococcus epidermidis*[36], whereas the methanol extract of this plant was reported to inhibit the growth of *S. aureus, B. subtilis* and *E. coli*[37].

The aqueous extract from the leaves of *S. alba* displayed an antimicrobial activity against both tested Gram-negative bacteria (*E. coli* and *P. aeruginosa*), one (*M. luteus*) of the three tested Gram-positive bacteria (*M. luteus*), and the yeast *C. albicans*. In agreement with this is that both the methanol and ethyl acetate extracts have been reported to have antimicrobial activity against Gram-positive (*Bacillus cereus* and *S. aureus*) and Gram-negative (*E. coli*) bacteria [38], but the inhibition activity against *P. aeruginosa* and *C. albicans* is newly reported here.

Although the results of this work are consistent with those reported by Tao *et al.*[39] in that the ethanol extract of *S. caseolaris* had no antimicrobial activity against *B. subtilis, S. aureus*, *P. aeruginosa* and *E. coli*, our ethanol extract did inhibit the growth of *E. coli*. In addition, we found that the crude water extract of *S. caseolaris* leaves could inhibit the growth of *C. albicans*, which is consistent with the report that not only the ethanol leaf extract but also the methanol extract of the cork of this plant could inhibit *C. albicans*[40]. Thus, these two species from the family *Sonneratiaceae* merit further investigation for antimicrobial agents.

Finally, the ethanol extract from the leaves of *A. occidentale* demonstrated antimicrobial activity against *E. coli* and *P. aeruginosa*, which is similar to that previously reported [41-43], but is in contrast with the previous report that the ethanol extract of this plant’s leaves also inhibited *M. luteus*, *S. aureus* and *B. subtilis*[41,42], which was not observed in the present study.

Overall, the ethanol and aqueous extracts exhibited potential antimicrobial activity and merit further bioactivity guided fractionation to obtain the bioactive component(s). However, since the crude hexane and DCM extracts could not be screened, their initial fractionation and assay for antimicrobial activity may be of value, with further bioactivity guided fractionation as required.

## Conclusion

The *in vitro* cytotoxic, antioxidant, lipase inhibitory and antimicrobial activities of 52 species of Thai medicinal plants were studied using the tissue source(s) that are used in traditional folk medicine. The crude hexane extract from the vines of *B. strychnifolia* was found to exert a strong *in vitro* cytotoxic activity against human cancer cell lines and certainly merits further enrichment to isolate the potentially promising bioactive component(s). This work provides the basic data base of selected Thai medicinal plants species that can be used to identify potential novel bioactive compounds for pharmacological and phytochemical investigations. However, the other parts of each plant may not be used in folk medicine for reasons other than a lack of bioactive compound(s), such as digestibility, taste or presence of other deleterious components, and so the other tissues of positive plants should be screened. Regardless, the simple approach of using the knowledge of folk medicine as a first guide to select plants for pharmacological screening appears validated, although it should be extended to include not only the other tissue parts of positive plants, but also close phylogenetic relatives and cultivars.

## Abbreviations

CM: Complete media; DCM: Dichloromethane; DMSO: Dimethyl sulfoxide; DPPH: 2,2-diphenyl-1-picryhydrazyl; EC_50_: Half maximal effective concentration; FBS: Fetal bovine serum; IC_50_: Half maximal inhibitory concentration; MIC: Minimum inhibitory concentration; MTT: 3-(4,5-dimethylthiazol-2-yl)-2,5-diphenyltetrazolium bromine; *p*-NPL: *para*-nitrophenyllaurate; PPL: Porcine pancreatic lipase; SD: Standard deviation.

## Competing interests

The authors declare that they have no competing interests.

## Authors' contributions

This is the original work of the authors and the manuscript was not previously submitted to BITE. All authors are aware of, and accept responsibility for, the manuscript. Conceived and designed the experiments: CK, PW, PP, TY, WA. Performed the experiments: CK. Analyzed the data: CK, PW, PP, TY, WA. Contribute reagents, materials, analysis tools: KL, CS, PW, PP, JS, WA. Wrote the paper: CK, PW, TY, WA. All authors read and approved the final manuscript.

## Pre-publication history

The pre-publication history for this paper can be accessed here:

http://www.biomedcentral.com/1472-6882/12/217/prepub

## Supplementary Material

Additional file 1 **Table S1.** Ethnobotanical data and percent yield of the investigated Thai plant extracts. **S2***In vitro* cytotoxic activity of crude extracts against human cancer cell lines. **S3***In vitro* antimicrobial activity and minimal inhibitory concentrations (MIC) of the investigated crude extracts. (DOC 174 kb) Click here for file
