# Supplementary material for: Studies of the in vitro cytotoxic, antioxidant, lipase inhibitory and antimicrobial activities of selected Thai medicinal plants
Source: BMC Complement Altern Med. 2012 Nov 13;12:217. doi: 10.1186/1472-6882-12-217 (PMC3519510; doi:10.1186/1472-6882-12-217)
Supplement: Additional file 1 — Table S1. Ethnobotanical data and percent yield of the investigated Thai plant extracts. S2In vitro cytotoxic activity of crude extracts against human cancer cell lines. S3In vitro antimicrobial activity and minimal inhibitory concentrations (MIC) of the investigated crude extracts. (DOC 174 kb) [file 1472-6882-12-217-S1.doc]

**Table 1** Ethnobotanical data and percent yield of the investigated Thai plant extracts

| **Plant species** | **Family** | **Part tested** | **Traditional uses**^[29]^ | **Extract yield (%)** | | | |
| --- | --- | --- | --- | --- | --- | --- | --- |
|  |  |  |  | **Extracts** | | | |
|  |  |  |  | **Hexane** | **DCM** | **Ethanol** | **Water** |
| *Acrostichum aureum* Linn. | Pteridaceae | Leaves | Rheumatism | 2.2 | 5.6 | 19.5 | 9.2 |
| *Acanthus ebracteatus* Vahl. | Acanthaceae | Leaves | Rheumatism and snake bites | 2.6 | 1.6 | 19.3 | 5.6 |
| *Acanthus ilicifolius* Linn. | Acanthaceae | Leaves | Diabetes, diuretic, dyspepsia, hepatitis, leprosy, neuralgia, paralysis, ringworms, rheumatism, skin diseases, snake bites and stomach pains | 0.3 | 0.7 | 3.6 | 7.5 |
| *Allium sativum* Linn. | Alliaceae | Bulbs | Wounds, ulcers, skin infections, flu, athlete's foot, some viruses, strep, worms, respiratory ailments, high blood pressure, blood thinning, cancer of the stomach, colic, colds, kidney problems, bladder problems and ear aches | 9.7 | 10.0 | 9.7 | 9.7 |
| *Anacardium occidentale* Linn. | Anacardiaceae | Leaves | Antiseptic, antidysenteric, antibacterial, ulcers and astringent | 1.8 | 0.6 | 1.8 | 3.4 |
| *Avicennia alba* Bl. | Avicenniaceae | Leaves | Antifertililty, skin disease, tumor and ulcers | 1.4 | 4.3 | 12.6 | 7.4 |
| *Avicennia officinalis* Linn. | Avicenniaceae | Leaves | Aphrodisiac, diuretic and hepatitis | 5.5 | 3.4 | 38.2 | 18.2 |
| *Azima sarmentosa* (Blume) Benth.  *Barleria lupulina* Lindl. | Salvadoraceae  [Acanthaceae](http://th.wikipedia.org/wiki/Acanthaceae) | Leaves  Whole plant | Unknown  Anti-inflammatory for insect bites | 0.4  3.5 | 1.8  2.2 | 6.3  3.2 | 8.4  6.4 |
| *Barringtonia asiatica* (Linn.) Kurz | Lecythidaceae | Leaves | Stomachache and rheumatism | 9.6 | 2.1 | 20.4 | 10.3 |
| *Bauhinia strychnifolia* Craib. | Caesalpiniaceae | Leaves  Vines | Antipyretic | 2.6  1.1 | 1.1  1.8 | 12.1  13.5 | 3.4  2.6 |
| *Bruguiera sexangula* Poir. | Rhizophoraceae | Leaves | Antitumor | 3.1 | 4.6 | 18.4 | 8.6 |
| *Celosia argentea* Linn. | [Amaranthaceae](http://en.wikipedia.org/wiki/Amaranthaceae) | Flower | Antioxidant , inflammation, antivirus and antibacterial | 1.5 | 1.1 | 6.1 | 25.9 |
| *Centella asiatica* Urban. | Umbelliferae | Leaves | Hypertension, diarrhea and urinary tract infections | 9.2 | 9.9 | 6.9 | 9.7 |
| *Ceriops tagal* (Perr.) C.B.Rob | Rhizophoraceae | Leaves | Unknown | 1.4 | 3.3 | 6.4 | 10.2 |
| *Clerodendrum inerme* Gaertn. | Verbenacceae | Leaves | Antiseptic, arrests bleeding, asthma, hepatitis, ringworm, stomach pains and uterine stimulant | 1.8 | 4.2 | 12.6 | 9.2 |
| *Coccoloba uvifera* (L.) Jacq. | Polygonaceae | Leaves | Throat ailments and dysentery | 0.4 | 1.3 | 5.6 | 4.2 |
| *Colocasia esculenta* (L.) Schott var. aquatilis Hassk. | [Araceae](http://en.wikipedia.org/wiki/Araceae) | Roots | Colorectal cancer and digestive disorders | 1.4 | 4.6 | 3.6 | 8.3 |
| **Table 1 (Continued)** | | | | | | | |
| **Plant species** | **Family** | **Part tested** | **Traditional uses**^[29]^ | **Extract yield (%)** | | | |
|  |  |  |  | **Extracts** | | | |
|  |  |  |  | **Hexane** | **DCM** | **Ethanol** | **Water** |
| *Coscinium fenestratum* (Gaertn.) Colebr. | Menispermaceae | Stem | Ophthalmopathy, wounds, inflammations, ulcers, skin diseases, abdominal, disorders, jaundice, diabetes, tetanus, fever and general debility | 0.5 | 1.1 | 1.0 | 1.0 |
| *Derris trifoliata* Lour. | Leguminosae | Leaves | Arrests hemorrhage, antispasmodic and stimulant | 0.3 | 2.2 | 10.2 | 7.4 |
| *Dolichandrone spathacea* (L.f.) K. Schum. | Bignoniaceae | Leaves | Antitumor and antiseptic | 3.1 | 6.9 | 24.9 | 7.7 |
| *Eurycoma longifolia* Jack. | Simaroubaceae | Roots | Malaria, aches, persistent fever, dysentery, glandular swelling, bleeding (as a coagulant), edema, hypertension, syphilitic sores and ulcers | 0.0 | 0.5 | 0.3 | 1.9 |
| *Excoecaria agallocha* Linn. | Euphorbiaceae | Leaves | Epilepsy, conjunctivitis, dermatitis, haematuria, leprosy and a purgative | 1.1 | 0.3 | 7.3 | 3.7 |
| *Flagellaria indica* Linn. | Flagellariaceae | Leaves | Traditional shampoo | 1.3 | 2.7 | 30.8 | 10.7 |
| *Ipomoea batatas* Lamk. | Convulvulaceae | Roots | Tonic during pregnancy and to induce lactation | 1.2 | 3.4 | 5.1 | 10.4 |
| *Kalanchoe pinnata* Pers. | [Crassulaceae](http://en.wikipedia.org/wiki/Crassulaceae) | Leaves | Treat ailments such as infections, rheumatism and inflammation | 1.2 | 1.9 | 20.1 | 9.6 |
| *Lumnitzera littorea* Voigt. | Combretaceae | Leaves | Headaches, boils, ulcers and diarrhea | 2.6 | 3.6 | 10.2 | 25.4 |
| *Lumnitzera racemosa* Willd. | Combretaceae | Leaves | Antifertility, asthma, snake bite and diabetes | 4.5 | 3.6 | 2.6 | 18.1 |
| *Mangifera foetida* Lour. | Anacardiaceae | Leaves | Antipyric | 8.8 | 3.4 | 30.6 | 18.4 |
| *Melientha suavis* Pierre. | Opiliaceae | Leaves | Neoplasm, cardiovascular diseases, inflammation, neurodegenerative pathologies, cataracts, diabetes and anti-aging process | 2.8 | 2.7 | 7.7 | 9.9 |
| *Momordica cochinchinensis* Spreng. | Cucurbitaceae | Seed salve | Relief of dry eyes | ND | ND | 31.1 | 3.7 |
| *Moringa oleifera* Lam. | Moringaceae | Leaves  Seeds | Anti-hyperlipidemic, anti-inflammatory and antioxidant | 2.0  16.4 | 0.7  0.3 | 2.0  0.9 | 8.5  3.4 |
| *Murdania iroiformis* (Hassk.) Rolla Rao et Kammathy | Commelinaceae | Leaves | Chronic bronchitis and cancer | 9.7 | 9.9 | 9.2 | 9.4 |
| *Nypa fruticans* Wurmb. | Palmae | Leaves | Asthma, diabetes, leprosy, rheumatism and snake bite | 2.9 | 2.1 | 5.8 | 10.4 |
| *Orthosiphon grandiflorus* (Blume) Miq. Bolding. | Labiatae | Whole plant | Treatment of arthritis, gout and rheumatism | 4.0 | 1.1 | 0.8 | 4.8 |
| *Peperomia pellucida* ( L.) Humb; Bonpl & Kunth | [Piperaceae](http://en.wikipedia.org/wiki/Piperaceae) | Leaves | Treating abdominal pain, abscesses, acne, boils, colic, fatigue, gout, headache, renal disorders and rheumatic joint pain | 4.5 | 2.3 | 8.9 | 12.9 |
| *Pereskia grandifolia* Haw. | [Cactaceae](http://en.wikipedia.org/wiki/Cactaceae) | Leaves | Cancer, high blood pressure, diabetes and diseases associated with rheumatism and inflammation | 2.4 | 1.3 | 30.3 | 12.4 |
| **Table 1 (Continued)** |  |  |  |  |  |  |  |
| **Plant species** | **Family** | **Part tested** | **Traditional uses**^[29]^ | **Extract yield (%)** | | | |
|  |  |  |  | **Extracts** | | | |
|  |  |  |  | **Hexane** | **DCM** | **Ethanol** | **Water** |
| *Phyllanthus amarus* Schum&Thonn. | Euphorbiaceae | Leaves | Astringent, cooling, diuretic, stomachic, febrifuge and antiseptic | 1.4 | 2.2 | 10.6 | 12.4 |
| *Pluchea indica* (Linn.) Less. | Compositae | Leaves | Fever, gangrenous ulcers and rheumatism | 0.2 | 0.2 | 5.8 | 6.5 |
| *Pseuderatherum platiferum* (Nees) Radlk. | Acanthaceae | Leaves | Diarrhea, diabetes and cancer | 4.5 | 3.4 | 18.6 | 9.3 |
| *Rhizophora apiculata* Blume. | Rhizophoraceae | Leaves | Hepatitis | 3.2 | 2.1 | 12.4 | 7.9 |
| *Rhizophora mucronata* Poir. | Rhizophoraceae | Leaves | Hepatitis | 3.4 | 2.7 | 16.3 | 12.1 |
| *Sandoricum koetjape* Burm.f. Merr. | [Meliaceae](http://en.wikipedia.org/wiki/Meliaceae) | Bark | Cancer | 2.6 | 4.3 | 15.1 | 23.3 |
| *Sesuvium portulacastrum* Linn. | Aizoaceae | Leaves | Hepatitis | 0.3 | 0.3 | 4.3 | 10.1 |
| *Sonneratia alba* j. Smith. | Sonneratiaceae | Leaves | Poultice in swellings and sprains | 4.1 | 2.6 | 15.4 | 7.3 |
| *Sonneratia caseolaris* Gaerth. | Sonneratiaceae | Leaves | Cough | 3.2 | 2.9 | 11.6 | 8.2 |
| *Solanum torvum* Swartz. | Solanaceae | Leaves | Colds and cough, pimples, skin diseases and leprosy | 2.5 | 0.5 | 13.0 | 8.7 |
| *Solanum trilobatum* Linn. | Solanaceae | Flower  Leaves | Cough | 2.5  5.3 | 1.1  2.3 | 2.1  2.9 | 5.8  3.3 |
| *Suaeda maritima* (L.) Dumort. | Chenopodiaceae | Leaves | Hepatitis | 1.3 | 0.8 | 10.5 | 4.2 |
| *Thespesia populnea* (L.) Soland ex Corr. | Malvaceae | Leaves | Inflammation and swollen joints | 4.7 | 5.4 | 30.1 | 24.3 |
| *Trichosanthes cucumerina* Linn. | Cucurbitaceae | Fruits | Inflammation | 1.6 | 0.8 | 0.6 | 1.9 |
| *Vernonia cinerea* Less. | Asteraceae | Whole plant | Colds and fever, cough dysentery, hepatitis, neurasthenia, furunculosis, snake bites, body ache, dizziness and hypertension | 0.2 | 0.3 | 0.5 | 11.1 |
| Most of the information is taken from [29] and the rest is from native Thai people.  ND; Not determined because of the limited solubility | | | | | | | |

**Table 2** *In vitro* cytotoxic activity of crude extracts against human cancer cell lines

| Plant species | Cell lines | | | | | | | | | | | | | | | |
| --- | --- | --- | --- | --- | --- | --- | --- | --- | --- | --- | --- | --- | --- | --- | --- | --- |
|  | A549 | | | | MDA-MB-231 | | | | KB3-1 | | | | SW480 | | | |
|  | IC_50_ (µg/mL)^a^ | | | | IC_50_ (µg/mL)^a^ | | | | IC_50_ (µg/mL)^a^ | | | | IC_50_ (µg/mL)^a^ | | | |
|  | **Hexane** | **DCM** | **Ethanol** | **Water** | **Hexane** | **DCM** | **Ethanol** | **Water** | **Hexane** | **DCM** | **Ethanol** | **Water** | **Hexane** | **DCM** | **Ethanol** | **Water** |
| *B. strychnifolia.*(Vine) | 5.95 ± 0.10 | **1.16 ± 0.12** | 16.62 ± 1.11 | > 100 | **0.28 ± 0.01** | 14.94 ± 3.27 | > 100 | > 100 | 1.63 ± 0.21 | **1.86 ± 0.15** | > 100 | 68.50 ± 3.00 | 7.25 ± 1.23 | 41.25 ± 4.53 | 19.00 ± 3.53 | > 100 |
| *C. fenestratum* | > 100 | 42.50 ± 10.11 | > 100 | > 100 | > 100 | 15.13 ± 7.19 | 40.00 ± 7.29 | > 100 | > 100 | **3.25 ± 0.24** | **5.15 ± 1.2** | > 100 | > 100 | 17.45 ± 4.37 | > 100 | > 100 |
| *E. longifolia* | 80.00 ± 17.90 | 16.00 ± 1.30 | 5.50 ± 1.10 | 50.00 ± 8.60 | 10.40 ± 0.10 | **1.60 ± 0.40** | **1.20 ± 0.20** | 24.30 ± 6.50 | 35.14 ± 10.50 | 13.12 ± 1.58 | 15.78 ± 2.03 | 45.65 ± 11.50 | 52.14 ± 10.54 | 32.14 ± 15.58 | 21.15 ± 7.25 | > 100 |
| *K. pinnata* | 35.00 ± 5.36 | 18.33 ± 4.15 | 15.00 ± 6.32 | 34.17 ± 7.66 | 87.50 ± 12.58 | 4.32 ± 0.31 | 3.18 ± 0.04 | > 100 | 7.53 ± 1.04 | **2.18 ± 0.23** | **1.03 ± 0.07** | > 100 | 44.06 ± 4.22 | **5.23 ± 0.98** | **6.12 ± 2.14** | > 100 |
| Positive control | Etoposide = 0.95 ± 0.06 µg/mL | | | | Doxorubicin = 0.48 ± 0.02 µg/mL | | | | Vinblastine = 1.13 ± 0.10 µg/mL | | | | Oxaliplatin = 3.56 ± 0.62 µg/mL | | | |

^a^Data are shown as the mean + 1 SD and are derived from three independent determinations. Figures in bold font represent the highest activity/activities against that cell line and are referred to in the text.

**Table 3** *In vitro* antimicrobial activity and minimal inhibitory concentrations (MIC) of the investigated crude extracts

| Plant species / Antimicrobial agent | Gram-positive bacteria | | | | | | | | | | | | Gram-negative bacteria | | | | | | | | Fungal strain | | | |
| --- | --- | --- | --- | --- | --- | --- | --- | --- | --- | --- | --- | --- | --- | --- | --- | --- | --- | --- | --- | --- | --- | --- | --- | --- |
|  | *Staphylococcus aureus* | | | | *Bacillus subtilis* | | | | *Micrococcus luteus* | | | | *Escherichia coli* | | | | *Pseudomonas aeruginosa* | | | | *Candida albicans* | | | |
|  | Inhibition zone*  (mm) | | MIC  (µg/mL) | | Inhibition zone*  (mm) | | MIC  (µg/mL) | | Inhibition zone*  (mm) | | MIC  (µg/mL) | | Inhibition zone*  (mm) | | MIC  (µg/mL) | | Inhibition zone*  (mm) | | MIC  (µg/mL) | | Inhibition zone*  (mm) | | MIC  (µg/mL) | |
|  | Ethanol | Water | Ethanol | Water | Ethanol | Water | Ethanol | Water | Ethanol | Water | Ethanol | Water | Ethanol | Water | Ethanol | Water | Ethanol | Water | Ethanol | Water | Ethanol | Water | Ethanol | Water |
| *A. occidentale* | – | – | – | – | – | – | – | – | – | – | – | – | 15 | – | 250 | – | 13 | – | 500 | – | – | – | – | – |
| *C. fenestratum* | 10 | – | 500 | – | 15 | – | 500 | – | 13 | – | 500 | – | 10 | – | 500 | – | – | – | – | – | 10 | – | 500 | – |
| *S. alba* | – | – | – | – | – | – | – | – | – | 13 | – | 500 | – | 10 | – | 250 | – | 10 | – | 125 | – | 10 | – | 250 |
| *S. caseolaris* | – | – | – | – | – | – | – | – | – | – | – | – | 15 | 13 | 500 | 250 | – | – | – | – | – | 15 | – | 125 |
| Chloramphenicol | 30 | | 7.81 | | 30 | | 31.3 | | 24 | | 31.3 | | 30 | | 31.3 | | 15 | | 125 | |  |  |  |  |
| Amphotericin B |  | |  | |  | |  | |  | |  | |  | |  | |  | |  | | 17 | | 250 | |

* 2 mg crude extract was used in each antimicrobial activity

- No inhibitory activity detected
